# Bird’s Eye View feature selection for high-dimensional data

**DOI:** 10.1038/s41598-023-39790-3

**Published:** 2023-08-16

**Authors:** Samir Brahim Belhaouari, Mohammed Bilal Shakeel, Aiman Erbad, Zarina Oflaz, Khelil Kassoul

**Affiliations:** 1 Division of Information and Computing Technology, College of Science and Engineering, Hamad Bin Khalifa University, Doha, Qatar; 2 Department of Industrial Engineering, Faculty of Engineering and Natural Sciences, KTO Karatay University, Konya, Turkey; 3 Geneva School of Economics and Management (GSEM), University of Geneva, 1211 Geneva, Switzerland

**Keywords:** Computer science, Mathematics and computing

## Abstract

In machine learning, an informative dataset is crucial for accurate predictions. However, high dimensional data often contains irrelevant features, outliers, and noise, which can negatively impact model performance and consume computational resources. To tackle this challenge, the Bird’s Eye View (BEV) feature selection technique is introduced. This approach is inspired by the natural world, where a bird searches for important features in a sparse dataset, similar to how a bird search for sustenance in a sprawling jungle. BEV incorporates elements of Evolutionary Algorithms with a Genetic Algorithm to maintain a population of top-performing agents, Dynamic Markov Chain to steer the movement of agents in the search space, and Reinforcement Learning to reward and penalize agents based on their progress. The proposed strategy in this paper leads to improved classification performance and a reduced number of features compared to conventional methods, as demonstrated by outperforming state-of-the-art feature selection techniques across multiple benchmark datasets.

## Introduction

The increasing number of high-dimensional datasets in various organizations is driving the need for advanced data mining techniques^1,2^. However, handling high-dimensional data presents a challenge that limits the application of data mining algorithms. To overcome this, feature selection^3^ and extraction methods are used to reduce the dimensions of the data. While feature extraction transforms raw data into a new feature space, feature selection algorithms choose the optimal subset of features from the raw data, leading to lower dimensionality and improved interpretability while preserving the actual data space^4^.

With the rise of high-dimensional data in various organizations, the need for effective feature selection algorithms has become increasingly crucial. Currently, several search mechanisms exist, including ranking-based methods^5^, swarm intelligence/evolutionary algorithms^6^, forward/backward search^7,8^, and nature-inspired meta-heuristics^9^. These approaches can be further classified as supervised^10^, semi-supervised^11^, or unsupervised^12^ based on the availability of training data labels. Despite their successes, supervised-wrapper configurations of these methods often face limitations in handling high-dimensional data. In this paper, we introduce the Bird's Eye View (BEV) model for feature selection that incorporates the strengths of supervised evolutionary algorithms in a wrapper configuration while addressing their limitations in high-dimensional data spaces.

**Figure 1:**
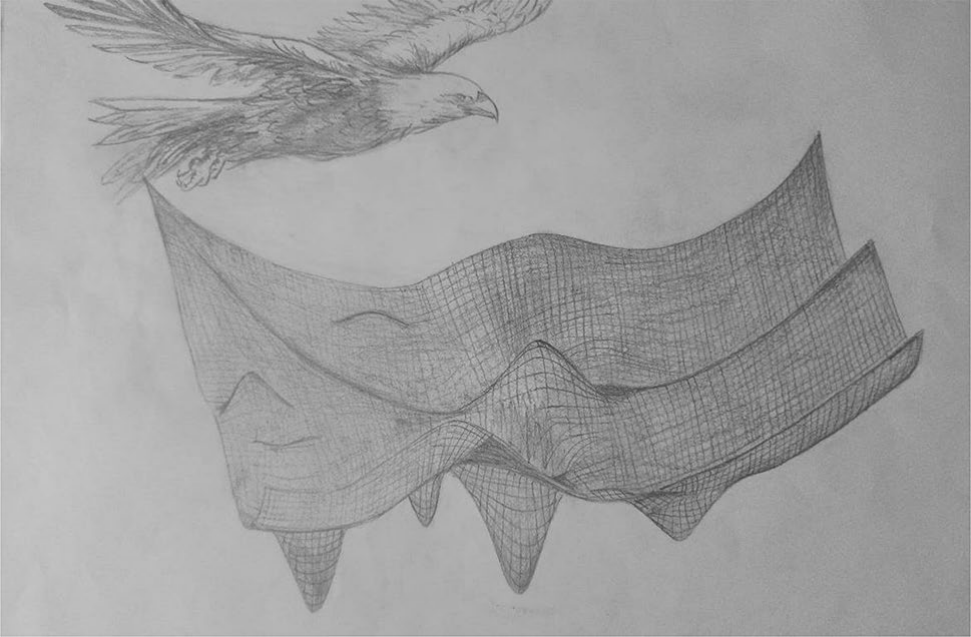
Eagle at a high altitude seeking the optimum way.

The BEV model draws inspiration from various natural mechanisms to achieve a comprehensive perspective on feature selection (as illustrated in Fig. 1). Similar to how a bird surveys a vast terrain to search for food from a high altitude, the BEV technique scours high-dimensional datasets for valuable features. Furthermore, the BEV approach resembles the biological process of gene regulation, in which a cell selects which genes to activate from its genome to form a unique gene pattern that enables each cell type to perform its specific function. This integration of nature-inspired mechanisms allows the BEV model to have a more comprehensive view of feature selection.

Our method determines which features to retain for optimal performance and discards unnecessary features. This resembles a reward-based training approach, similar to teaching a dog the desired behavior through positive reinforcement with treats, play, and other incentives. Our model's agents evaluate the performance of various subsets of data and reward improved performance with increased probabilities. Conversely, reduced performance results in lower probabilities.

The proposed BEV model is a unique feature selection technique with the following significant contributions:

1. The design of the Markov chain and Reinforcement learning paradigms in an evolutionary framework for efficient communication between search agents and optimal global solution.
2. The evolution of agents is based on the Markov chain, generating new agents with improved accuracy and associated probabilities.
3. A new metric for evaluating classifiers is proposed as a fitness function.
4. The movement of agents in search space is guided by reinforcement learning, rewarding progress and penalizing regress with changes in associated probabilities.
5. The process involves iterations that result in improved agents and reduced computational complexity by restricting the number of agents involved in each iteration.
6. The recursive approach includes choosing a subset of characteristics at each stage in order to remove unimportant features while keeping important ones.

## Background and literature review

In recent years, various optimization techniques have been developed to tackle complex problems across fields such as computer science, engineering, finance, machine learning, and data science. This section reviews three of the most prominent algorithms: Markov Chain, Evolutionary Algorithms (specifically Genetic Algorithm), and Reinforcement Learning. These methods have proven to be effective in addressing challenging optimization problems and have been widely used. Despite their importance, these methods have certain drawbacks, including constrained exploration, the necessity for parameter modification, the inability to handle multiple objectives, and slow or premature convergence. Thus, it is crucial to take these restrictions into account when applying them to challenging optimization problems. One can overcome these limitations by carefully characterizing the problem, selecting the best algorithm, fine-tuning the parameters, and using complementary strategies to solve the shortcomings of each approach. In the following subsections, a brief overview of each approach, its key concepts, applications, advantages, and usage in the proposed work are provided.

### Markov chain

The Markov analysis is a technique for estimating the value of a variable that is solely dependent on its current state, without taking into account prior activity^13^. It calculates a random variable based on the present state of other variables using a probability matrix. This makes it a useful tool for evaluating state transitions in various fields such as surveillance^14^, machine learning^15^, and computer vision^16^. Its popularity is due to its ease of use and good prediction accuracy, often outperforming more complex models^17^. Although widely used, few studies have applied it to feature extraction^18–20^, where Markov chain features are extracted to capture dynamic changes in data and used by learning algorithms to make decisions. A new concept of feature selection, based on the transition probabilities of the Markov chain, is proposed as an alternative to feature extraction in our work.

### Evolutionary algorithms

An Evolutionary Algorithm (EA) is a computational method that solves problems by mimicking the behavior of living organisms using nature-inspired mechanisms^21^. The use of EAs for feature selection has received significant attention in academia, with various algorithms being proposed, including Particle Swarm Optimization (PSO)^22–24^, Genetic Algorithm (GA)^25,26^, Artificial Bee Colony (ABC)^27^, Genetic Programming (GP)^28^, Gravitational Search Algorithm (GSA)^29^ and Ant Colony Optimization (ACO)^30,31^. One advantage of EAs is their population-based search approach, which involves a team of entities exploring the fitness landscape to find the globally optimum solution. This allows for more effective and efficient exploration of vast and challenging search areas. The sharing of information among team members also enables the discovery of potential regions of the search space and the narrowing of the search to critical areas. Additionally, these methods balance exploration and exploitation, allowing for faster convergence while avoiding local optimal solutions. These unique characteristics make EAs a promising approach for designing neural networks^32^.

Genetic algorithms are the type of evolutionary algorithms used in this work. A genetic algorithm is an optimization technique that uses a process inspired by natural evolution to find the best solution for a problem. The algorithm works by iteratively searching through a space of potential solutions, selecting and breeding the most promising candidates based on a set of rules inspired by genetics, and introducing random mutations to create new solutions. This process is repeated until either a satisfactory solution is found or a specified number of iterations have passed. Genetic algorithms are commonly used in machine learning and data analysis to find optimal model parameters^33–35^ or identify patterns in data^36,37^. The same approach is applied to feature selection in the proposed work. Initially, a set of possible feature combinations is generated randomly, represented as pairs. These pairs are then evaluated using a fitness function that assigns a score based on their accuracy. The pairs with the highest scores are selected for reproduction, mimicking the process of natural selection. The process repeats until a satisfactory solution is found or a specified number of iterations have been reached.

### Reinforcement learning

Reinforcement learning^38,39^ is a method of learning by interacting with the environment and learning from rewards received from actions taken. It aims to find the best long-term solution by balancing exploration and exploitation. This type of learning has a lot of potential for effective feature selection in the subspace of features. Feature selection can be performed through single-agent^40,41^ or multi-agent^42^ decision processes. In a single-agent process, only one agent decides on the selection or deselection of features, resulting in a large action space and the risk of getting stuck in a local optimum solution. On the other hand, in a multi-agent process, multiple agents are involved in feature selection, which enables easier exploration and convergence of the search space. This approach also resembles natural systems, as there are similarities between reinforcement learning and biological systems^43^.

## A fitness function to better evaluation of classifiers

Classifier evaluation metrics^44,45^ are used to determine the effectiveness of a classification model by comparing the predicted outcomes to the actual outcomes. Some commonly used metrics for evaluating classifiers include:

- *Accuracy* It measures the percentage of correct predictions made by the model out of all predictions. It is defined as $\left( {TP + TN} \right)/\left( {TP + TN + FP + FN} \right)$, where TP (True Positives) represents the number of positive instances correctly predicted, TN (True Negatives) represents the number of negative instances correctly predicted, FP (False Positives) represents the number of negative instances incorrectly predicted as positive, and FN (False Negatives) represents the number of positive instances incorrectly predicted as negative.
- *Precision* It is the ratio of true positive predictions to the sum of true positive and false positive predictions. Precision measures the ability of the classifier to avoid false positive predictions and is defined as $TP/\left( {TP + FP} \right)$.
- *Recall (Sensitivity or True Positive Rate)* It is the ratio of true positive predictions to the sum of true positive and false negative predictions. Recall measures the ability of the classifier to detect positive instances and is defined as $TP/\left( {TP + FN} \right)$.
- *F1-Score* It is the harmonic mean of precision and recall, used to balance precision and recall when they are in conflict. The *F1*-Score is defined as $\left( {2 \cdot {\text{Precision}} \cdot {\text{Recall}}} \right)/\left( {{\text{Precision }} + {\text{ Recall}}} \right)$. It provides a balance between precision and recall, as it is a measure of the harmonic mean of these two values.
- *AUC-ROC curve* The receiver operating characteristic (ROC) curve plots the true positive rate against the false positive rate at different classification thresholds. The area under the ROC curve (AUC) summarizes the performance of the classifier.
- *Confusion matrix* It is a table used to evaluate the performance of a classification algorithm, by comparing the predicted classes to the actual classes.
- *Log Loss (Cross-Entropy Loss)* It measures the performance of a classification model by calculating the likelihood of the predicted outcomes being accurate.

The choice of evaluation metric will depend on the problem and the goals of the classifier. For example, precision may be important when false positive predictions are costly, while recall may be important when false negative predictions are costly. Note that in multiclass classification, precision, recall, and *F1*-Score can be calculated for each class and then averaged using macro-average or micro-average methods. The confusion matrix is a table that has *C* rows and *C* columns, where *C* is the number of classes. Each row of the matrix represents the instances in a predicted class, while each column represents the instances in an actual class. For example, consider a multiclass classification problem with *C* = 3 classes. The confusion matrix would be a 3 × 3 table, as shown below in Table 1.

**Table 1:** Confusion matrix.

	Actual class 1	Actual class 2	Actual class 3
Predicted class 1	$TP_{1}$	$FP_{12}$	$FP_{13}$
Predicted class 2	$FP_{21}$	$TP_{2}$	$FP_{23}$
Predicted class 3	$FP_{31}$	$FP_{32}$	$TP_{3}$

Where $TP_{i}$ represents the number of instances of class *i* that are correctly predicted as class *i*, and $FP_{ij}$ represents the number of instances of class *j* that are incorrectly predicted as class *i*.

From the values in the confusion matrix, various evaluation metrics such as accuracy, precision, recall, and *F1*-Score for each class, as well as macro-average and micro-average across all classes, can be calculated. The choice of evaluation metric will depend on the problem and the goals of the classifier.

In this study, a new metric is proposed to better monitor the performance of classifiers. Our new metric will accurately measure the accuracy of each class and is suitable for use in feature selection. Therefore, this metric can be used as a fitness function in our search algorithm

1 $$ \mathop {\min }\limits_{i} \left( {\frac{{TP_{i} }}{{TP_{i} + \mathop \sum \nolimits_{j \ne i} FP_{ij} }}} \right) $$

## Methods

The goal of feature selection is to identify and select the smallest possible subset of relevant features from a larger set of features, to improve the accuracy, interpretability, and computational efficiency of the model. The idea is to remove redundant, irrelevant, and noisy features that may negatively impact the model's performance. The selection of a smaller set of relevant features not only aids in mitigating overfitting but also enhances the interpretability and comprehensibility of the model for human experts. A new tree search algorithm is developed in this paper to better explore the search space representing all the possible subsets. Our algorithm starts from the root node and expands it to generate child nodes until a goal node is found.

The search algorithm begins with a randomly selected subset of features represented by a sequence of 1 s and 0 s, where 1 s indicate selected features and 0 s indicate unselected features, i.e., each leaf belongs to $\left\{ {0,1} \right\}^{d}$, where the integer $d$ is the size of the total features.

The root leaf generates ${\mathcal{A}}$ new subsets, known as children, by randomly altering the states of each pair of features. The children are formed using the transition probability of the Markov chain of each feature pair, the transition matrices reflect the likelihood of transitioning between distinct states {00, 01, 10, 11}, with initial values for the transition probabilities of 0.25.

Through the expansion, the transition matrices are updated based on a rewards function reflecting the performance of the generated children. Therefore, each new leaf generated will inherit the transition matrices of each pair of features from the parent and update them according to the concept of reward that will describe later in this section.

Updating these transition matrices in the right manner will favor certain extensions of the proposed tree to better explore the search space. After each cycle or iteration, only the highest-performing leaves are kept for further expansion.

The following definitions are crucial for a thorough explanation of the approach:

- States or leaves are defined in: $\left\{ {0,1} \right\}^{d}$, where the integer $d$ is the size of the total features.
- ${\mathcal{A}}$: number of children generated by each leaf; each offspring represents a subset of selected features.
- ${\mathcal{M}}_{{\mathcal{A}}}$: number of top-performing leaves that are selected for further expansion at each iteration.
- *t*: number of iterations.
- *s*: number of stages.
- ${\mathcal{F}}_{j}^{t,s}$: represents the status of the *j*th leaf (i.e., state) at time *t* and stage *s*, ${\mathcal{F}}_{j}^{t,s} \in \left\{ {0,1} \right\}^{d} ,$ *j* = $1, \ldots , {\mathcal{M}}_{{\mathcal{A}}}$, which specifies whether each feature has been selected or not. The position of values of 1 shows the location of the features that have been chosen, and the position of the values of 0 indicates the position of the features that have been eliminated.
- $f_{i,j}^{t,s} :$ represents the value of the *i*th feature in the *j*th leaf at time $t$ and stage $s$, $f_{i,j}^{t,s} \in \left\{ {0,1} \right\}$, $i = 1,2, \ldots ,d$ and *j* = $1, \ldots , {\mathcal{M}}_{{\mathcal{A}}}$ .
- $$C_{i,j}^{t,s} : $$ represents the state of *i*th feature pair, $$C_{i,j}^{t,s} = \{ f_{2i - 1,j}^{t,s} ,$$ $$f_{2i,j}^{t,s} \}$$, at time $$t$$ and stage $$s$$ of *j*th leaf.
- $P_{i,j}^{t - 1,s} ({ \mathcal{C}}_{i,j}^{t.s} |{ }{\mathcal{C}}_{i,j}^{t - 1,s} { })$: transition probability from the pair ${\mathcal{C}}_{i,j}^{t - 1,s} { }$ to the pair ${ \mathcal{C}}_{i,j}^{t.s}$, it represents the actions of the evolutional algorithm.
- $d$: dimension of data or number of features $f_{i,j}^{t,s} , i = 1,2, \ldots ,d$;
- *n*: number of observations of data.
- $$\varepsilon$$: reward function.

### Genetic algorithm

The BEV algorithm utilizes a smart branching evolution approach that is based on dynamic Markov chains. At each new expansion, a fixed number of leaves (${\mathcal{M}}_{{\mathcal{A}}}$) are chosen. Each leaf is represented by a sequence consisting of 1 s and 0 s and they are organized in pairs within the sequence. The process begins with a root leaf and generates ${\mathcal{A}}$ children leaves, where ${\mathcal{A}}$ is less than ${\mathcal{M}}_{{\mathcal{A}}}$. Since the number of generated leaves does not exceed ${\mathcal{M}}_{{\mathcal{A}}}$, all of them are selected. During the next expansion, each leaf (or child) generates ${\mathcal{A}}$ leaves, resulting in a total of ${\mathcal{A}} \cdot {\mathcal{A}}$ children and ${\mathcal{A}}$ parent leaves. These children and parent leaves are evaluated, and the best ${\mathcal{M}}_{{\mathcal{A}}}$ leaves are chosen for the expansion.

In the subsequent step, each leaf from the selected ${\mathcal{A}} \cdot {\mathcal{M}}_{{\mathcal{A}}}$ leaves generates a ${\mathcal{A}}$ child, resulting in (${\mathcal{A}} \cdot {\mathcal{M}}_{{\mathcal{A}}}$) children and ${\mathcal{M}}_{{\mathcal{A}}}$ parent leaves. Again, these leaves are assessed, and only the best ${\mathcal{M}}_{{\mathcal{A}}}$ leaves are selected for the next expansion. This process continues until there is no further improvement in the quality of the solution.

**Figure 2:**
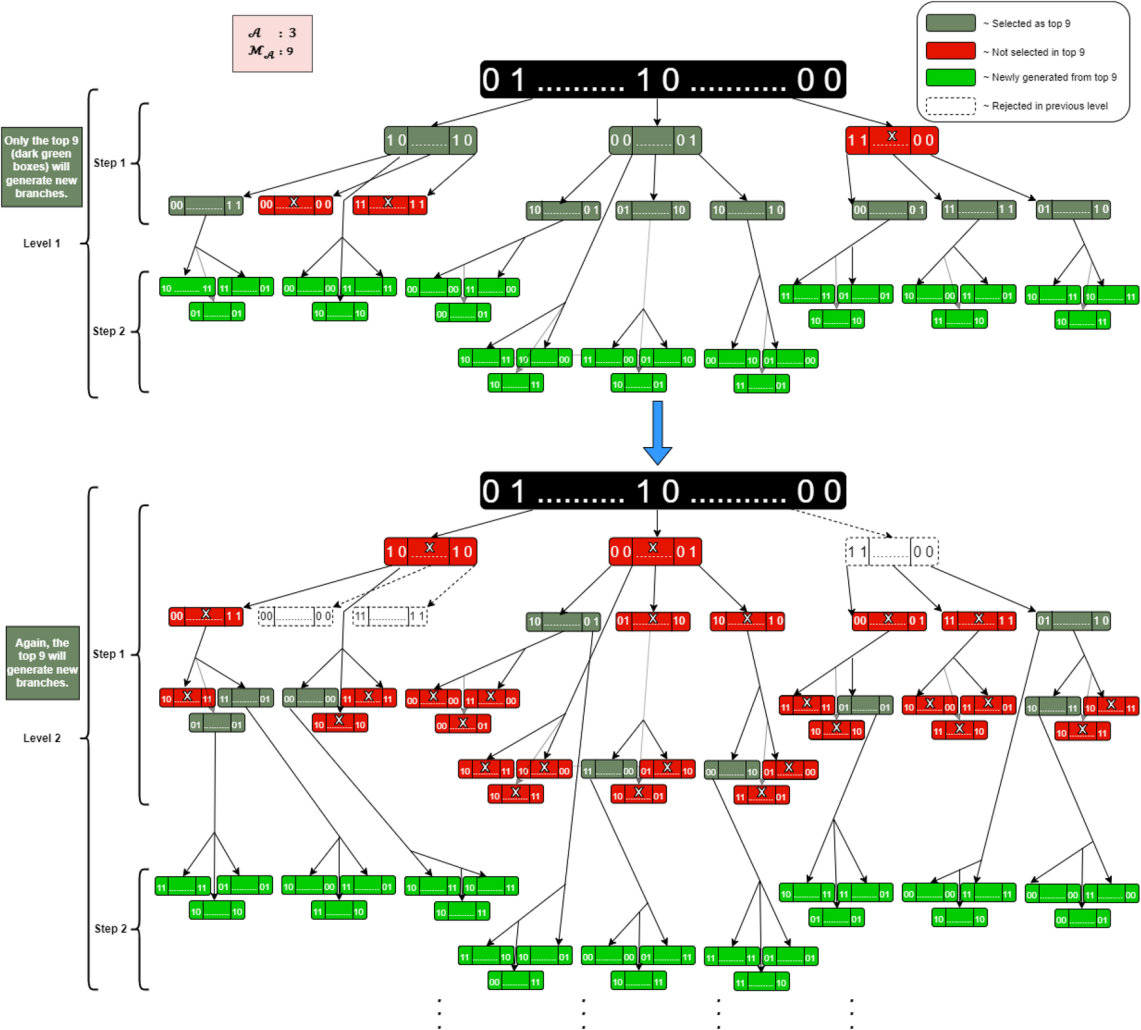
Process implementation in recursive levels. The process explains how the search space in an upcoming stage is reduced by considering only the best-performing features from the previous stage. We select or omit the specified features by assigning a 1 or 0 to each feature position.

Figure 2 illustrates the process of the BEV method, which involves expanding the children and selecting the most effective subset of features with ${\mathcal{A}}$ set to 3 and ${\mathcal{M}}_{{\mathcal{A}}}$ set to 9. Starting from the root leaf, three leaves are generated and all of them will be selected as they do not exceed the value ${\mathcal{A}} \cdot {\mathcal{M}}_{{\mathcal{A}}}$. The next expansion results in 9 children and 3 parent leaves, and the 9 best leaves will be chosen based on their performance (step 1). From the selected 9 leaves, a total of 27 leaves (children) are generated, leading to a combined set of 36 leaves (including parents and children). Similarly, in the next expansion, the 9 best leaves among the 36 will be chosen (step 2), and this process continues iteratively.

**Figure 3:**
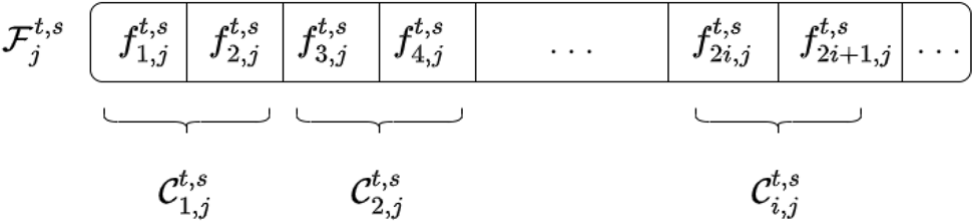
The features are gathered two by two in the leaf ${\mathbf{\mathcal{F}}}_{j}^{t,s}$.

**Figure 4:**
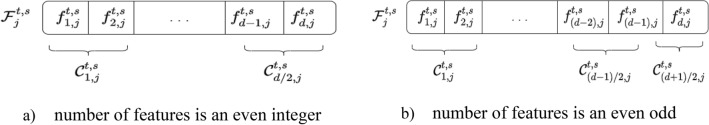
Dividing features into pairs.

Each leaf is represented by a sequence of 1 s and 0 s, where the features are grouped in pairs, as shown in Fig. 3. Every pair of features for each leaf has its transition matrix that determines the expansion process for that pair. Two scenarios must be taken into account when features are grouped two by two. Figure 4a, b demonstrate these two scenarios depending on whether the dimension *d* is even or odd.

### Markov decision process (MDP) and reinforcement learning

In order to determine the optimal subset of features that effectively differentiate between different classes, the BEV algorithm utilizes an smart approach to update transition probabilities during the transition from one state to another. This updating process is based on a reward and penalty mechanism. When the fitness function shows improvement, a reward value is added to the transition probability associated with the corresponding direction. At the same time, one third of the reward value is deducted from the transition probabilities of other directions. On the other hand, if the fitness function does not improve, a penalty value is applied to the transition probability of the relevant direction, while one third of the penalty value is added to the transition probabilities of other directions.

**Figure 5:**
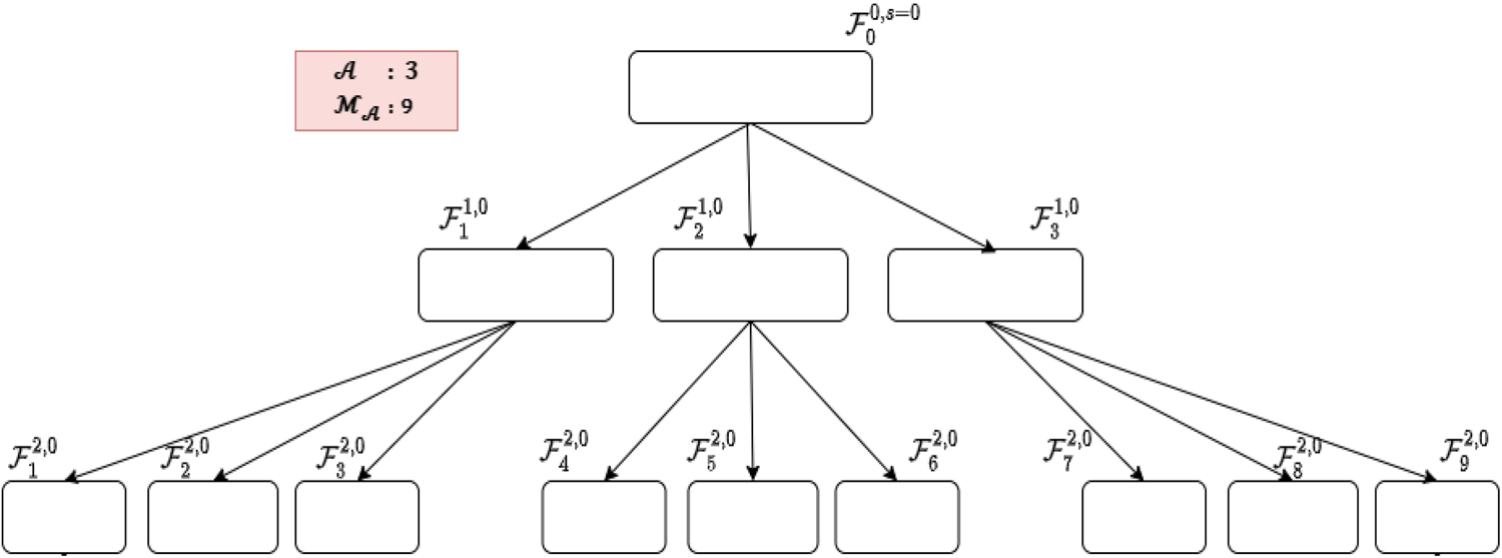
Process of expanding tree when  ${\mathcal{A}} = 3, {\mathcal{A}} \cdot {\mathcal{M}}_{{\mathcal{A}}}$ = 9.

**Figure 6:**
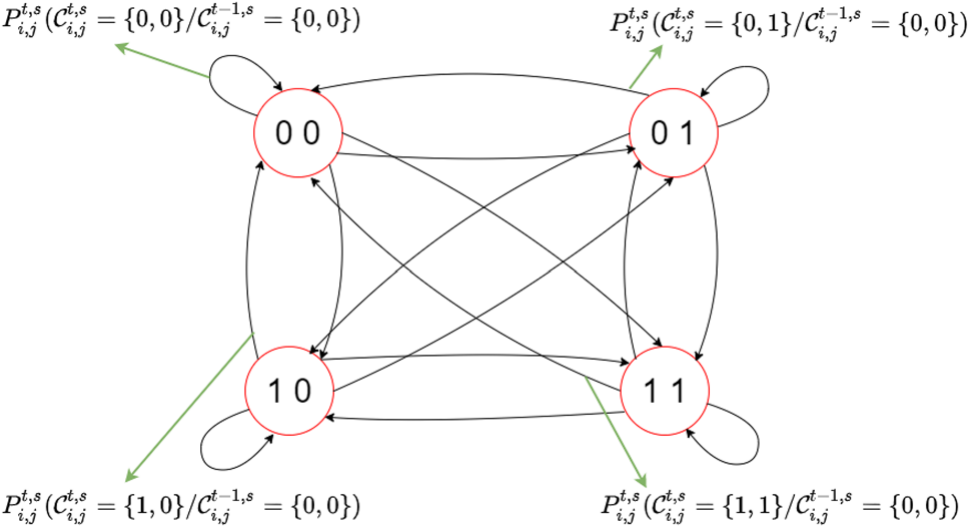
Dynamic Markov Chain for pairs.

As each Markov chain has four states {00, 01, 10, 11}, each pair of features at each leaf of ${\mathcal{F}}_{j}^{t,s}$ has four separate probability mass functions that govern the expansion process. Each child leaf will inherit these probability mass functions, or transition matrices, from the parent leaf and update them based on the fitness function as shown in Figs. 5 and 6.

The fitness function, denoted by $f$, can be interpreted as the classification accuracy at the state ${\mathcal{F}}_{j}^{t,s} ,$

2 $$ f:\left\{ {0,1} \right\}^{d} \to \left[ {0,1} \right] $$

The accuracy is calculated based solely on the features chosen with a value of 1 at their positions. The fitness function $f$ can be chosen as a minimum accuracy for each class as:

3 $$ \mathop {\min }\limits_{1 \le i \le K} \left( {\frac{{TP_{i} }}{{TP_{i} + \mathop \sum \nolimits_{j \ne i} FP_{ij} }}} \right) $$

where $TP_{i}$ represents the number of instances of class *i* that are correctly predicted as class *i*, and $FP_{ij}$ represents the number of instances of class *j* that are incorrectly predicted as class *i*. The value $K$ represents the total number of classes.

In the case where $${\mathcal{A}} =$$ 3 and $${\mathcal{A}} \cdot {\mathcal{M}}_{{\mathcal{A}}}$$ $$=$$ 9, Fig. 5 illustrates the early stages of expansion in a process, where three leaves, denoted as $${\mathcal{F}}_{j}^{t = 1,s = 0}$$ with $$j = 1, 2, 3, $$ emerge from the root leaf. Another 9 leaves are generated from the 3 leaves $${\mathcal{F}}_{j}^{t = 1,s = 0}$$ noted $${\mathcal{F}}_{j}^{t = 2,s = 0}$$ for $$j = 1$$ to 9. From these 12 leaves, only 9 are selected for continued expansion through the application of fitness functions, $$f\left( {{\mathcal{F}}_{j}^{t = 1,s = 0} { }} \right) $$ for $$j = 1, 2, 3$$ and $$f\left( {{\mathcal{F}}_{j}^{t = 2,s = 0} { }} \right) $$ for j = 1 to 9, which determines the most suitable leaves for growth.

The growth of each leaf is achieved through the transitions of each pair of features, represented by $C_{i,j}^{t,s}$ .The progression is guided by the transition probabilities, which are visualized in Fig. 6 through the presentation of four probability mass functions.

The transition probability of the *i*th pair at time *t* and stage *s* can be described as follows:

4 $$ P_{i,j}^{t - 1,s} ({ \mathcal{C}}_{i,j}^{t.s} |{ }{\mathcal{C}}_{i,j}^{t - 1,s} { }) = \left\{ {\begin{array}{*{20}l} {{\mathcal{P}}_{0,i,j}^{t - 1,s} } \hfill & {if\quad {\mathcal{C}}_{i,j}^{t,s} = \left\{ {0,0} \right\}} \hfill \\ {{\mathcal{P}}_{1,i,j}^{t - 1,s} } \hfill & { if\quad {\mathcal{C}}_{i,j}^{t,s} = \left\{ {0,1} \right\}} \hfill \\ {{\mathcal{P}}_{2,i,j}^{t - 1,s} } \hfill & { if\quad {\mathcal{C}}_{i,j}^{t,s} = \left\{ {1,0} \right\}} \hfill \\ {{\mathcal{P}}_{3,i,j}^{t - 1,s} } \hfill & { if\quad {\mathcal{C}}_{i,j}^{t,s} = \left\{ {1,1} \right\}} \hfill \\ \end{array} } \right. $$

5 $$ \mathop \sum \limits_{h = 0}^{3} {\mathcal{P}}_{h,i,j}^{t - 1,s} = 1 $$

6 $$ {\mathcal{C}}_{i,j}^{t - 1,s} \in \left\{ {\left\{ {0,0} \right\},\left\{ {0,1} \right\},\left\{ {1,0} \right\},\left\{ {1,1} \right\}} \right\} $$

**Figure 7:**
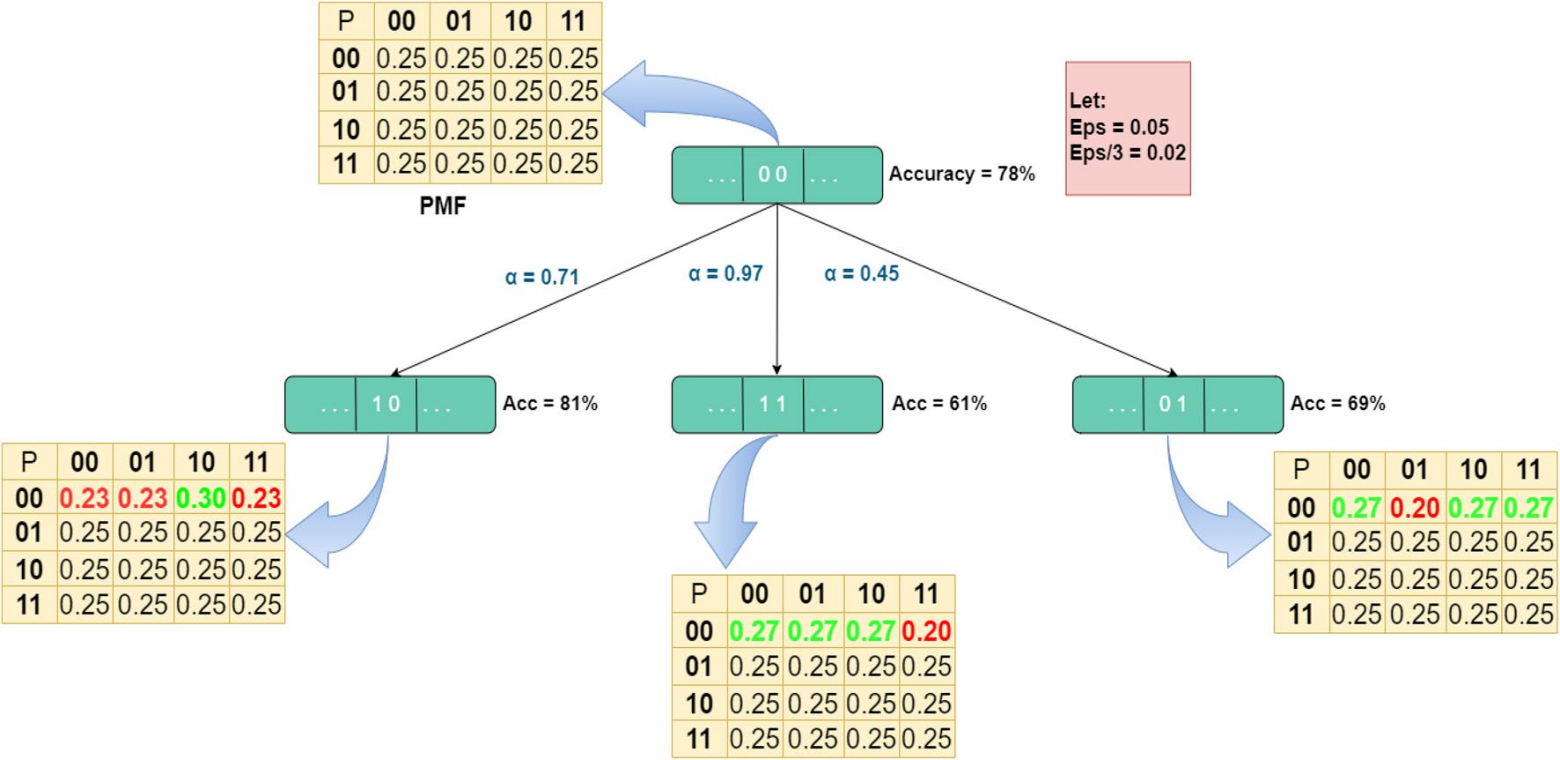
Probability updating mechanism based on the rewarding scheme by adding ${{\varvec{\upvarepsilon}}}$ to the appropriate direction as a reward and subtracting ${{\varvec{\upvarepsilon}}}$/3 to the other direction if the fitness function was improved and vice versa.

Figure 7 illustrates an example of how the probabilities are updated according to the fitness function values where it was initially supposed to be uniformly distributed, i.e., $P_{i,1}^{0,s} \left( {C_{i,0}^{t = 0,s = 0} } \right) = 0.25$. When the fitness function improves, a reward in the form of a value (ε) is added to the transition probability associated with the corresponding direction. Simultaneously, a deduction of ε/3 is made from the transition probabilities of other directions. Conversely, if the fitness function fails to improve, a penalty is applied by subtracting ε from the transition probability of the relevant direction, while ε/3 is added to the transition probabilities of other directions.

**Figure 8:**
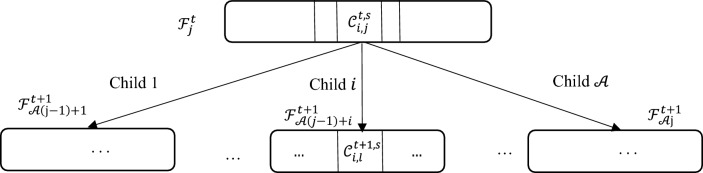
Expanding of the leave ${\mathbf{\mathcal{F} }}_{{\mathbf{j}}}^{{{\mathbf{t}},{\mathbf{s}}}}$ to ${\mathcal{A}}$ different children leaves.

Figure 8 clarifies the process of our approach, where each leaf ${\mathcal{F}}_{j}^{t,s}$ from ${\mathcal{A}} \cdot {\mathcal{M}}_{{\mathcal{A}}}$ leaves will be expanded to ${\mathcal{A}}$ leaves noted as follows:

7 $$ {\mathcal{F} }_{{{\mathcal{A}}\left( {j - 1} \right) + 1}}^{t + 1,s} , {\mathcal{F} }_{{{\mathcal{A}}\left( {j - 1} \right) + 2}}^{t + 1,s} , \ldots , {\mathcal{F} }_{{{\mathcal{A}}.j}}^{t + 1,s} $$

The selected best ${\mathcal{A}} \cdot {\mathcal{M}}_{{\mathcal{A}}}$ leaves, according to the fitness function, will be given new labels of ${\mathcal{F} }_{{\text{j}}}^{t + 1,s} {\text{for}} j = 1 {\text{to}} {\mathcal{M}}_{{\mathcal{A}}}$.

**Figure 9:**
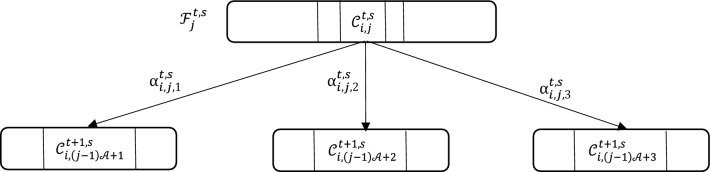
Expanding of the leaf ${\mathbf{\mathcal{F} }}_{{\mathbf{j}}}^{{{\mathbf{t}},{\mathbf{s}}}}$ to  ${\mathcal{A}}$ = 3 different children leaf where ${{\varvec{\upalpha}}} _{{{\mathbf{i}},{\mathbf{j}},{\mathbf{k}}}}^{{{\mathbf{t}},{\mathbf{s}}}}$ are generated from an independent identically uniform distribution between [0,1] to define new pairs in other leaves according to their p.m.f.

At each stage *s* and iteration *t*, new leaves are identified by generating ${\mathcal{A}}$ independent uniform random variables, denoted $\alpha_{j,i,r}^{t,s}$, for each leave *j* and each pair of features *i*. These variables are drawn from a uniform distribution between 0 and 1, with *r* = 1, …, ${\mathcal{A}}$, as illustrated in Fig. 9.

The transition pair from ${\mathcal{C}}_{i,j}^{t.s}$ to ${\mathcal{C}}_{i,\left(j-1\right)\mathcal{A}+r}^{t+1.s}$ is controlled by the values of the random variable ${\alpha }_{j,i,r}^{t,s}$ as indicated by Eq. (8).

8 $$ { \mathcal{C}}_{{i,\left( {j - 1} \right){\mathcal{A}} + r}}^{t + 1.s} = \left\{ {\begin{array}{*{20}l} {\left\{ {0,0} \right\}} \hfill & { if\quad \alpha_{i,j,r}^{t,s} < {\mathcal{P}}_{{0,i,\left( {j - 1} \right){\mathcal{A}} + r}}^{t,s} } \hfill \\ {\left\{ {0,1} \right\}} \hfill & {if\quad {\mathcal{P}}_{{0,i,\left( {j - 1} \right){\mathcal{A}} + r}}^{t,s} \le \alpha_{i,j,r}^{t,s} < {\mathcal{P}}_{{0,i,\left( {j - 1} \right){\mathcal{A}} + r}}^{t,s} + {\mathcal{P}}_{{1,i,\left( {j - 1} \right){\mathcal{A}} + r}}^{t,s} } \hfill \\ {\left\{ {1,0} \right\}} \hfill & {if\quad {\mathcal{P}}_{{0,i,\left( {j - 1} \right){\mathcal{A}} + r}}^{t,s} + {\mathcal{P}}_{{1,i,\left( {j - 1} \right){\mathcal{A}} + r}}^{t,s} \le \alpha_{i,j,r}^{t,s} < {\mathcal{P}}_{{0,i,\left( {j - 1} \right){\mathcal{A}} + r}}^{t,s} + {\mathcal{P}}_{{1,i,\left( {j - 1} \right){\mathcal{A}} + r}}^{t,s} + {\mathcal{P}}_{{2,i,\left( {j - 1} \right){\mathcal{A}} + r}}^{t,s} } \hfill \\ {\left\{ {1,1} \right\}} \hfill & {Elsewhere} \hfill \\ \end{array} } \right. $$

where $$r = 1,2, \ldots ,{ } {\mathcal{A}}.$$

**Figure 10:**
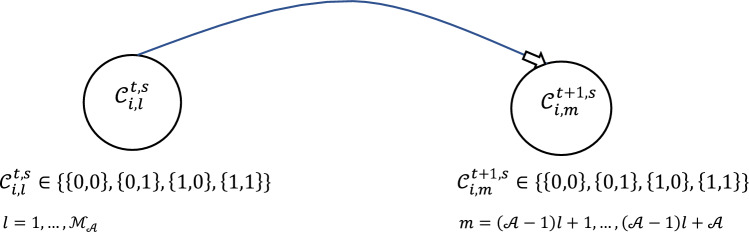
Transitions between pairs for the survival leaf and newly generated leaf.

At every expansion, one of the four probability mass functions for each pair of features for each leave generated from the ${\mathcal{A}} \cdot {\mathcal{M}}_{{\mathcal{A}}}$ leaves must be updated after inheriting the transition matrices from the parent leaf. This process is illustrated in Figs. 7, 8 and 10.

A probability mass function (p.m.f) is a function that describes the probability distribution of a discrete random variable. The following are some of the properties of a p.m.f that need to be kept during the process of updating:

- *Non-negativity* The p.m.f must be non-negative, meaning that it can take a value of 0, but it cannot be negative.
- *Non-exceeding 1* The p.m.f must be less than 1, meaning that it can take a value of 1, but it cannot be bigger.
- *Normalization* The sum of the p.m.f over all possible outcomes of the discrete random variable must equal 1, meaning that the probabilities of all outcomes add up to 100%.

Therefore, the procedure of probability of transition updating can be executed according to the following equation when the transition was performed from ${\mathcal{C}}_{i,j}^{t,s} = \left\{ {0,1} \right\}$ to ${\mathcal{C}}_{i,1}^{t,s} = \left\{ {1,1} \right\}$ for instance.

9 $$ {\text{P}}_{i,l}^{t + 1,s} \left( {x|{\mathcal{C}}_{i,j}^{t,s} = \left\{ {1,0} \right\}} \right) = \left\{ {\begin{array}{*{20}l} {{\mathcal{P}}_{0,i,l}^{t + 1,s} = b{\text{ max}}({\mathcal{P}}_{0,i,l}^{t,s} - \frac{\varepsilon }{3}\gamma ,0)} \hfill & {if\quad x = \left\{ {0,0} \right\}} \hfill \\ {{\mathcal{P}}_{1,i,l}^{t + 1,s} = b{\text{ max}}({\mathcal{P}}_{1,i,l}^{t + 1,s} - \frac{\varepsilon }{3}\gamma ,0)} \hfill & {if\quad x = \left\{ {0,1} \right\}} \hfill \\ {{\mathcal{P}}_{2,i,l}^{t + 1,s} = b{\text{ max}}({\mathcal{P}}_{2,i,l}^{t + 1,s} - \frac{\varepsilon }{3}\gamma ,0) } \hfill & {if \quad x = \left\{ {1,0} \right\}} \hfill \\ {{\mathcal{P}}_{3,i,l}^{t + 1,s} = b{\text{ min}}({\mathcal{P}}_{3,i,l}^{t + 1,s} + \varepsilon \gamma ,1)} \hfill & { if \quad x = \left\{ {1,1} \right\}} \hfill \\ \end{array} } \right. $$

where $\varepsilon$ is the value given by the reward function, $\gamma \in \left\{ { + 1, - 1} \right\},$ and

10 $$ b = \frac{1}{{{\text{max}}({\mathcal{P}}_{0,i,l}^{t,s} - \frac{\varepsilon }{3}\gamma ,0) + {\text{ max}}({\mathcal{P}}_{1,i,l}^{t + 1,s} - \frac{\varepsilon }{3}\gamma ,0) + {\text{ max}}({\mathcal{P}}_{2,i,l}^{t + 1,s} - \frac{\varepsilon }{3}\gamma ,0) + {\text{min}}({\mathcal{P}}_{3,i,l}^{t + 1,s} + \varepsilon \gamma ,1)}} $$

The other three probability mass functions ${\text{P}}_{i,l}^{t + 1,s} \left( {x|\left\{ {0,0} \right\}} \right),{\text{P}}_{i,l}^{t + 1,s} \left( {x|\left\{ {0,1} \right\}} \right),{\text{P}}_{i,l}^{t + 1,s} \left( {x|\left\{ {1,1} \right\}} \right)$ are kept the same.

The reward may be positive or negative depending on the evolution of the fitness function values from the leaf ${\mathcal{F}}_{m}^{t + 1,s}$ to the leaf ${\mathcal{F}}_{r}^{t,s}$, and it can be captured by the variable $\gamma$ as follows:

11 $$ \gamma = \left\{ {\begin{array}{*{20}l} { + 1 } \hfill & { if\quad f\left( {{\mathcal{F}}_{m}^{t + 1,s} } \right) > f\left( {{\mathcal{F}}_{r}^{t,s} } \right)} \hfill \\ { - 1 } \hfill & {Elsewhere } \hfill \\ \end{array} } \right. $$

The reward function $\varepsilon$ should be small variables depending on the progress of the fitness function, and different functions can be proposed as follows:

12 $$ \varepsilon \left( {fitness\left( {{\mathcal{F}}_{r}^{t,s} } \right) - fitness\left( {{\mathcal{F}}_{m}^{t + 1,s} } \right)} \right) =\upeta {*}\tanh \left| {fitness\left( {{\mathcal{F}}_{r}^{t,s} } \right) - fitness\left( {{\mathcal{F}}_{m}^{t + 1,s} } \right)} \right| $$

**Figure 11:**
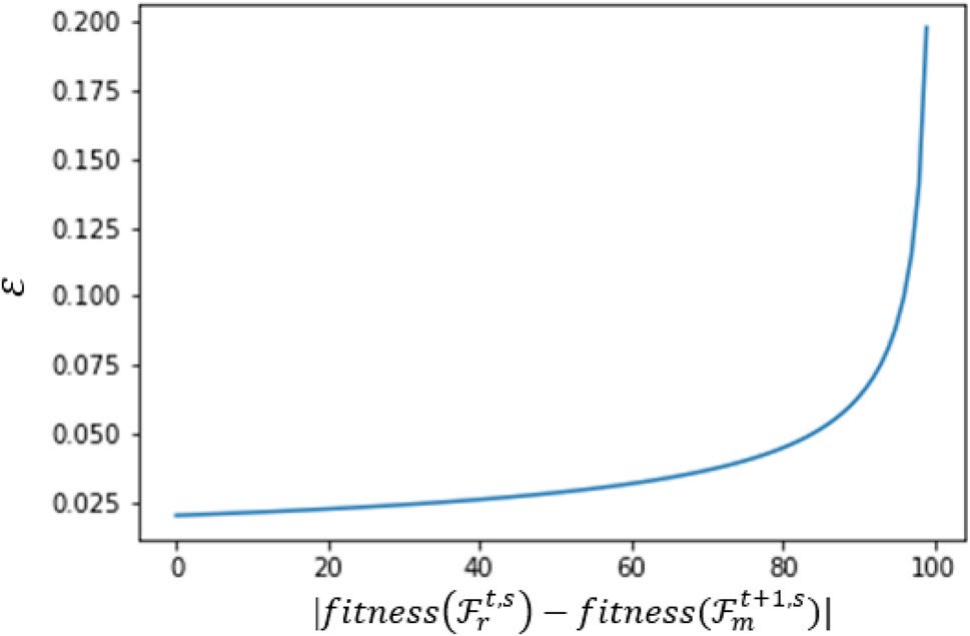
The reward function (${{\varvec{\upvarepsilon}}}$) plotted against the difference of fitness function of Eq. (13) when τ = 0.01, $\upeta$ = 0.2.

Or

13 $$ \varepsilon \left( {fitness\left( {{\mathcal{F}}_{r}^{t,s} } \right) - fitness\left( {{\mathcal{F}}_{m}^{t + 1,s} } \right)} \right) = \frac{\upeta }{{\sqrt {1 - \left| {fitness\left( {{\mathcal{F}}_{r}^{t,s} } \right) - fitness\left( {{\mathcal{F}}_{m}^{t + 1,s} } \right)} \right|} + \tau }} $$

where $\upeta$ and *τ* are two parameters that can be any small values, refer to Fig. 11.

**Figure 12:**
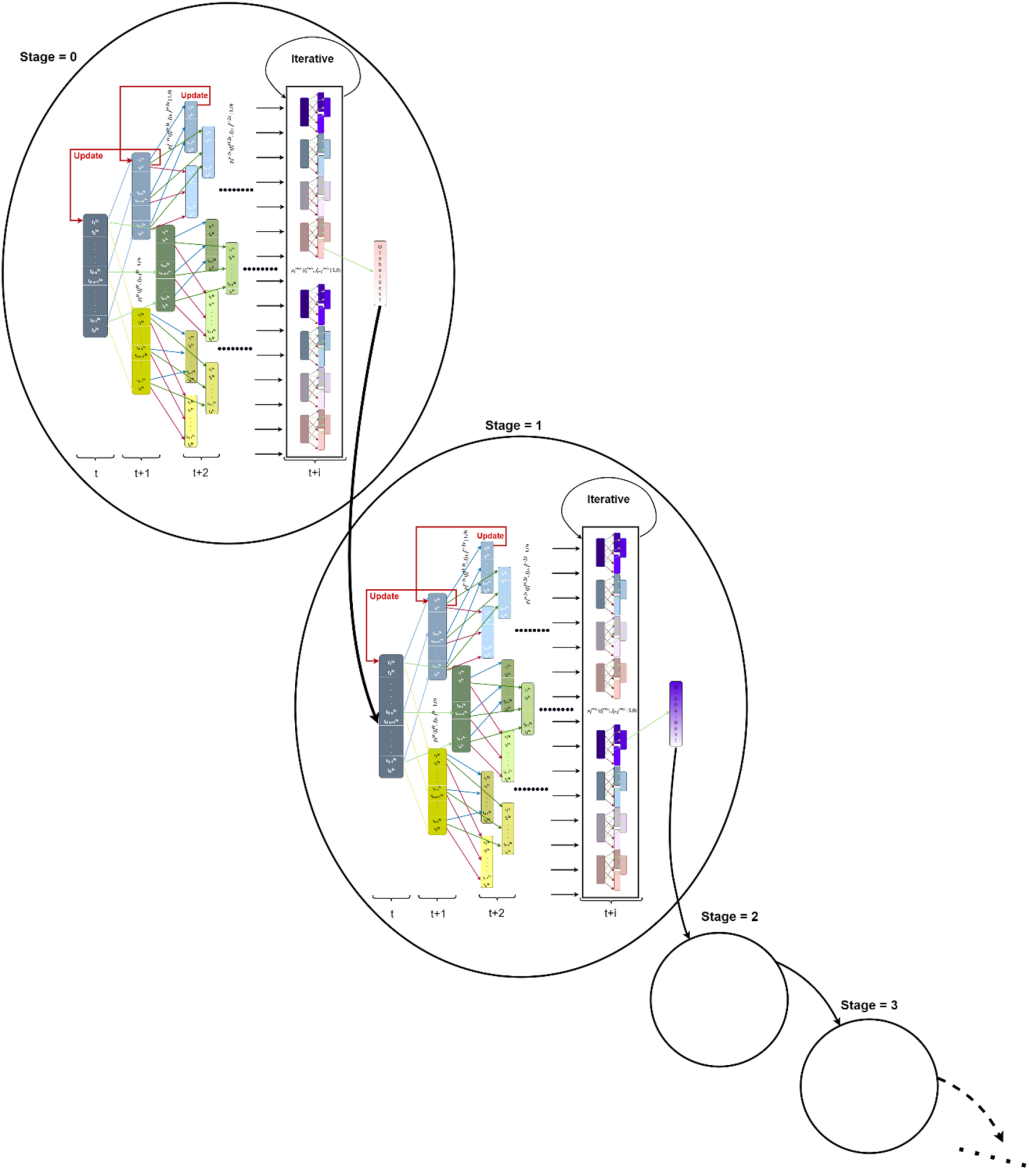
Progress through different stages.

The process proceeds through stages until accuracy can no longer be improved or further dimension reduction is not possible. The next stage (*s* + 1) will evaluate the best features selected from the previous stage (*s*) as the root of the new stage (*s* + 1). The progression through stages is necessary when there is a progression in performance, as shown in Fig. 12.

**Figure 13:**
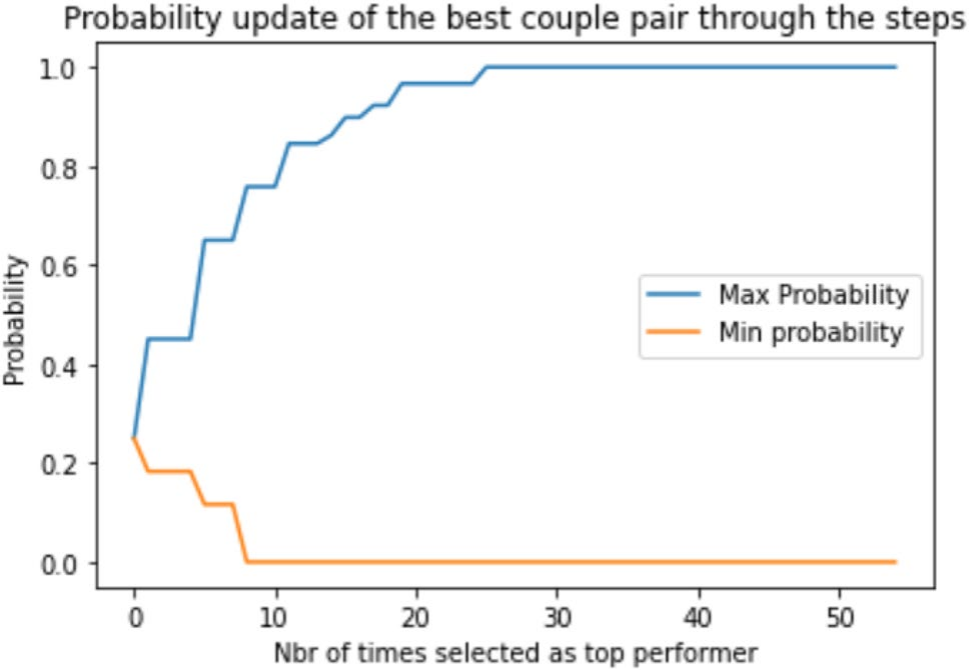
The evaluation of the transition probability of the best pair of features to determine when the equilibrium distribution will be attained.

As shown in Fig. 13, most transition probabilities will eventually converge to either 1 or 0, referred to as the equilibrium distribution, after a certain number of iterations determined by the reward value $\varepsilon$. At that point, it is necessary to reset the transition probabilities to 0.25 of the best leaf of the current stage as the root leaf for the next stage and repeat the branching process to see if higher accuracy can be achieved with fewer features.

**Figure 14:**
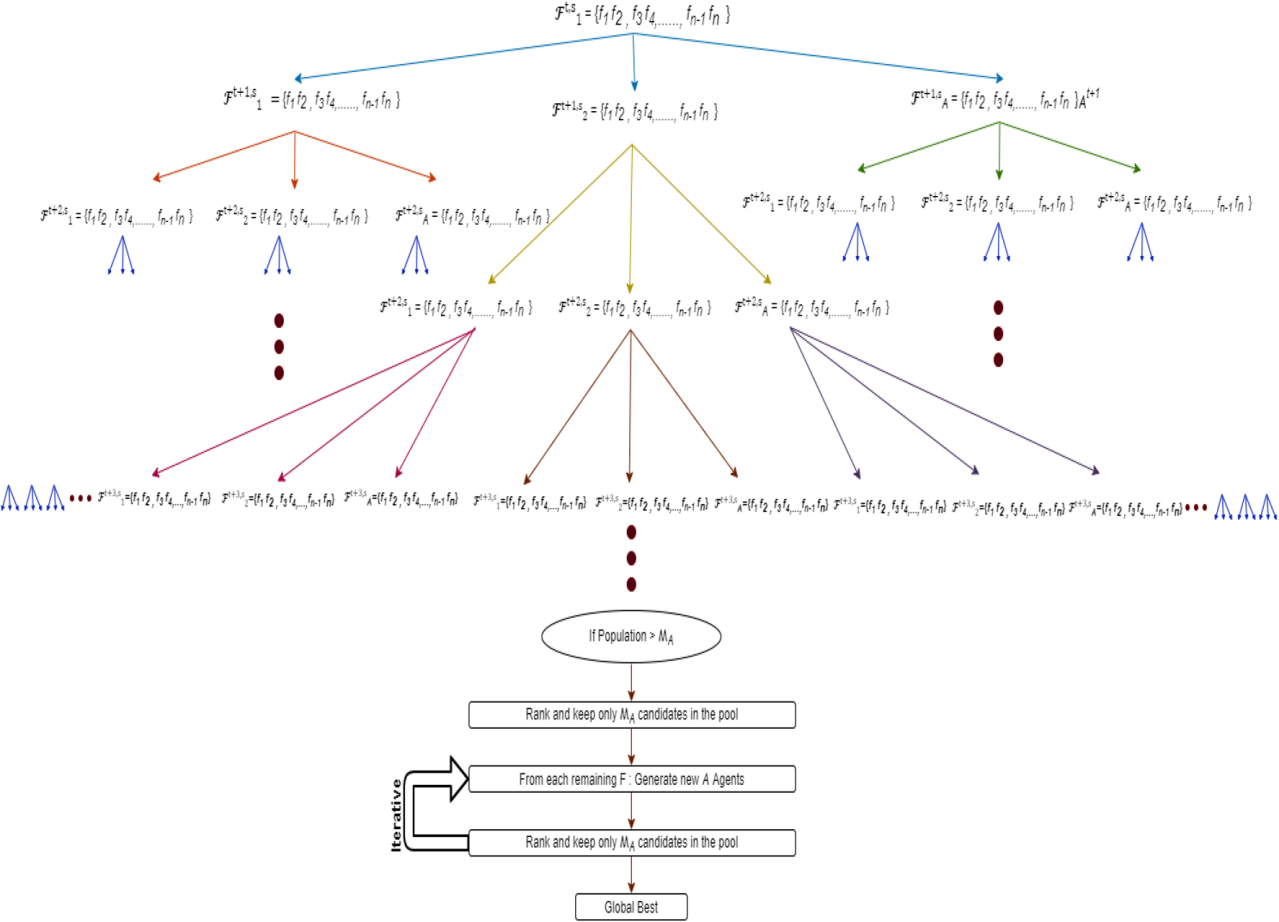
BEV feature selection process summary*.*

The overall structure of each stage of the BEV approach is summarized in Fig. 14.

## Results and discussion

This section plans to evaluate the proposed strategy by conducting experiments on a range of datasets that are commonly used for testing and comparison purposes. These datasets will serve as benchmarks to compare the performance with state-of-the-art methods and showcase the robustness of our technique. A thorough analysis of results, in terms of accuracy and size of selected features, will provide valuable insights into the strengths and weaknesses of our approach.

### Datasets

The evaluation of the suggested method was conducted using 10 real-world high-dimensional datasets. These datasets are used to test the performance of the method in terms of feature selection and classification tasks. The datasets used in the evaluation of the suggested method are gene expression datasets with high dimensionality, meaning there are more features than observations. Additionally, the datasets are challenging because of the imbalanced distribution of observations across classes. Table 2 provides information on the number of observations, number of features, and other relevant details for these datasets.

**Table 2:** Details of datasets.

Dataset	Observations	Features	Classes	% largest class	% smallest class	% sample distribution
Lung cancer	203	12,600	5	69	3	[69, 10, 10, 8, 3]
11 tumor	174	12,533	11	16	4	[16, $\ldots$,5,4]
Leukemia 2	72	11,225	3	39	28	[28, 33, 39]
Prostate	102	10,509	2	51	49	[49, 51]
Brain tumor 2	50	10,367	4	30	14	[14, 28, 30]
Brain tumor 1	90	5920	5	67	4	[67, 11, 11, 7, 4]
9 tumor	60	5726	9	15	3	[15, $\ldots$, 10, 10, 3]
DLBCL	77	5469	2	75	25	[75, 25]
Leukemia 1	72	5327	3	53	12	[53, 35, 12]
SRBCT	83	2308	4	35	13	[13, 22, 30, 35]

### Experimental settings

This paragraph describes the process of evaluating the proposed strategy using tenfold cross-validation. To account for the limited number of samples in the datasets, the cross-validation technique is used to create the training and test sets (no validation set is used). One-fold is reserved as the test set and not used in the feature selection process, while the remaining nine folds are used for building the training data. The selected features are then used to update the training and test sets, which are fed into the KNN algorithm to evaluate their performance. To ensure a fair and comprehensive assessment, each dataset is subjected to ten independent tenfold cross-validation tests with different random seeds, resulting in 100 total runs for each dataset. This approach aligns with previous research and provides a current assessment of the state-of-the-art^31,46^.

### Baseline methods

To demonstrate its effectiveness, the proposed work is compared with several existing feature selection algorithms that cover various techniques such as ant colony optimization, variable-length particle swarm optimization, comprehensive learning PSO with adaptive learning probability, and correlation-based feature selection. The comparison includes evolutionary models (TSHFS-ACO (two-stage hybrid feature selection model based on ant colony optimization)^31^, IRRF- SACO (Relevance-redundancy feature selection based on ant colony optimization)^47^), particle swarm optimization [Standard PSO, VL-PSO (Variable-Length Particle Swarm Optimization)^46^, CLPSO (Comprehensive Learning PSO) enhanced with the adaptive learning probability^48^, and CSO (Competitive Swarm Optimizer)^49^], graph-based [TFSACO (Text feature selection using ACO)^50^], and classical methods [LFS (linear forward selection), CFS (correlation-based feature selection)^51^, and FCBF (fast correlation-based feature selection)^52^].

### Parameter’ settings

Table 3 presents the parameters utilized in the proposed approach. The rest of the baseline methods compared are in line with those specified in prior studies^31,46^.

**Table 3:** Parameter’s settings.

Parameters	Settings
$\varepsilon$	$\upeta {*}\tanh \left| {fitness\left( {{\mathcal{F}}_{r}^{t,s} } \right) - fitness\left( {{\mathcal{F}}_{m}^{t + 1,s} } \right)} \right|$
$${\mathcal{A}}$$(# of new agents for every $$t + 1)$$	3
${\mathcal{M}}_{{\mathcal{A}}}$	9
Max $t$	36
KNN-$k$	5
$\eta$	0.2

### Results and discussion

**Figure 15:**
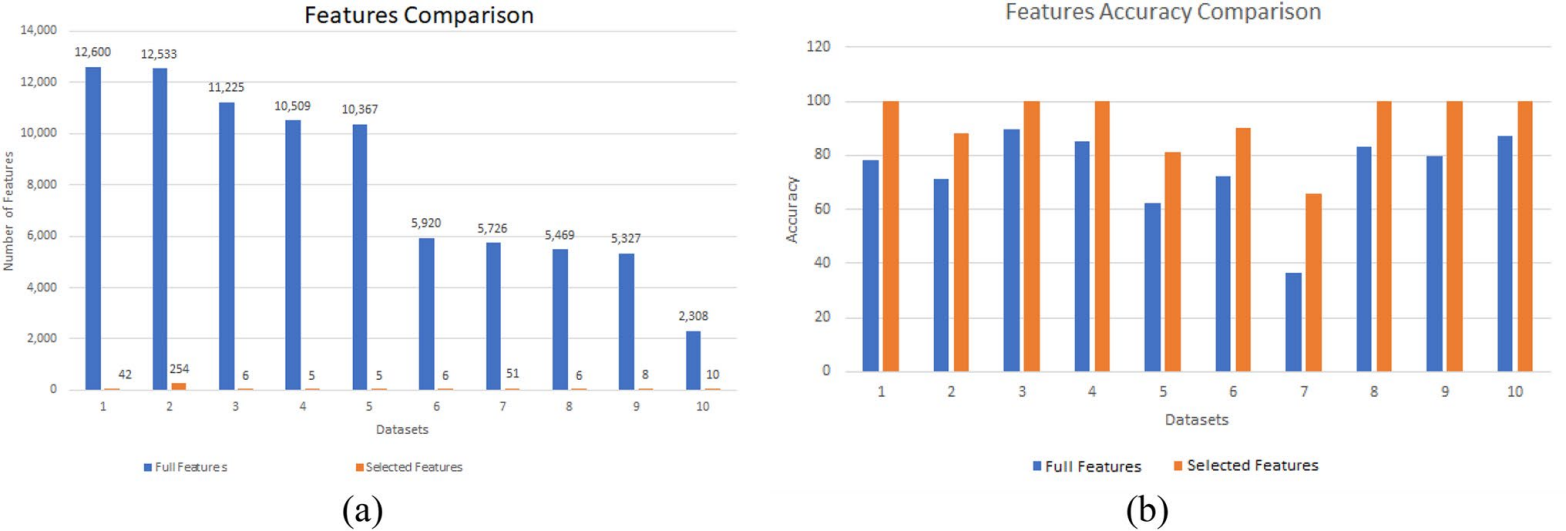
Performance comparison with the original feature vector of different datasets. (**a**) Performance in terms of dimensionality reduction. (**b**) Performance in terms of classification accuracy.

Table 4 demonstrates the performance of the proposed methodology on 10 high-dimensional real-world datasets. The comparison between the actual feature vector and the results of the proposed feature selection method is displayed for each dataset. The developed algorithm significantly improves classification accuracy and reduces the dimensionality of all datasets, as shown in Fig. 15. The graphical comparison highlights the improvement in the performance of the proposed feature selection results compared to the original feature vectors. Table 5 provides a detailed analysis of the performance of the proposed algorithm, including the best, worst, and meaningful results.

**Table 4:** Results on different datasets compared to the full feature set.

Dataset	Full feature set		Selected features	
	No. of features	Accuracy %	Average No. of features	Average accuracy %
Lung cancer	12,600	78.05	12.1	100.0
11 tumor	12,533	71.42	430.6	87.00
Leukemia 2	11,225	89.44	5.6	100.0
Prostate	10,509	85.33	6.4	100.0
Brain tumor 2	10,367	62.50	6.5	98.00
Brain tumor 1	5920	72.08	7.3	89.00
9 tumor	5726	36.67	108	64.80
DLBCL	5469	83.00	6.0	100.0
Leukemia 1	5327	79.72	6.1	100.0
SRBCT	2308	87.08	10.4	100.0

**Table 5:** Best, worst, and mean results on different datasets by the proposed algorithm.

Dataset	Accuracy (%)			Features			Stages		
	Best	Worst	Mean $\pm$ std	Best	Worst	Mean	Min	Max	Average
Lung cancer	100.0	100.0	100 $\pm$ 0.00	6	26	12.1	8	15	12
11 tumor	90.0	80.00	87.00 $\pm$ 3.49	59	1521	430.6	3	8	5.5
Leukemia 2	100.0	100.0	$100 \pm 0.00$	2	8	5.6	10	15	11.9
Prostate	100.0	100.0	$100 \pm 0.00$	4	13	6.4	10	14	12
Brain tumor 2	100.0	80.00	$98.00 \pm {5}.{37}$	3	18	6.5	9	15	12.4
Brain tumor 1	89.0	89.00	$89.00 \pm 0.00$	2	26	7.3	9	12	10.7
9 tumor	77.77	55.55	$64.80 \pm 7.04$	5	355	108	4	12	8.1
DLBCL	100.0	100.0	$100 \pm 0.00$	3	14	6.0	10	13	10.5
Leukemia 1	100.0	100.0	$100 \pm 0.00$	4	10	6.1	9	13	10.9
SRBCT	100.0	100.0	$100 \pm 0.00$	3	26	10.4	7	12	9.1

**Figure 16:**
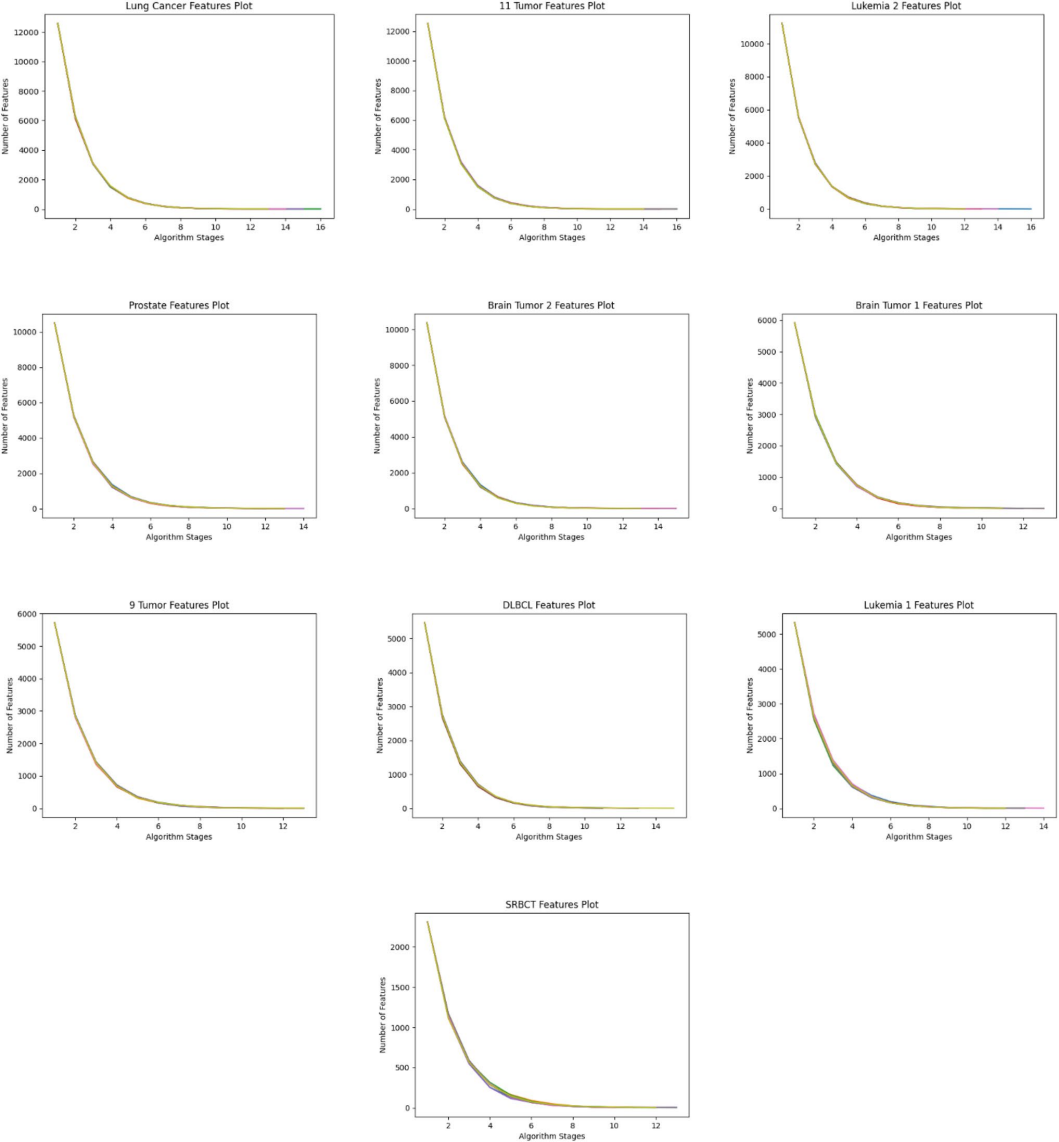
Performance of the proposed algorithm on ten different datasets over ten independent runs. The graphs show the performance in terms of reducing the number of dimensions with recursive stages.

**Figure 17:**
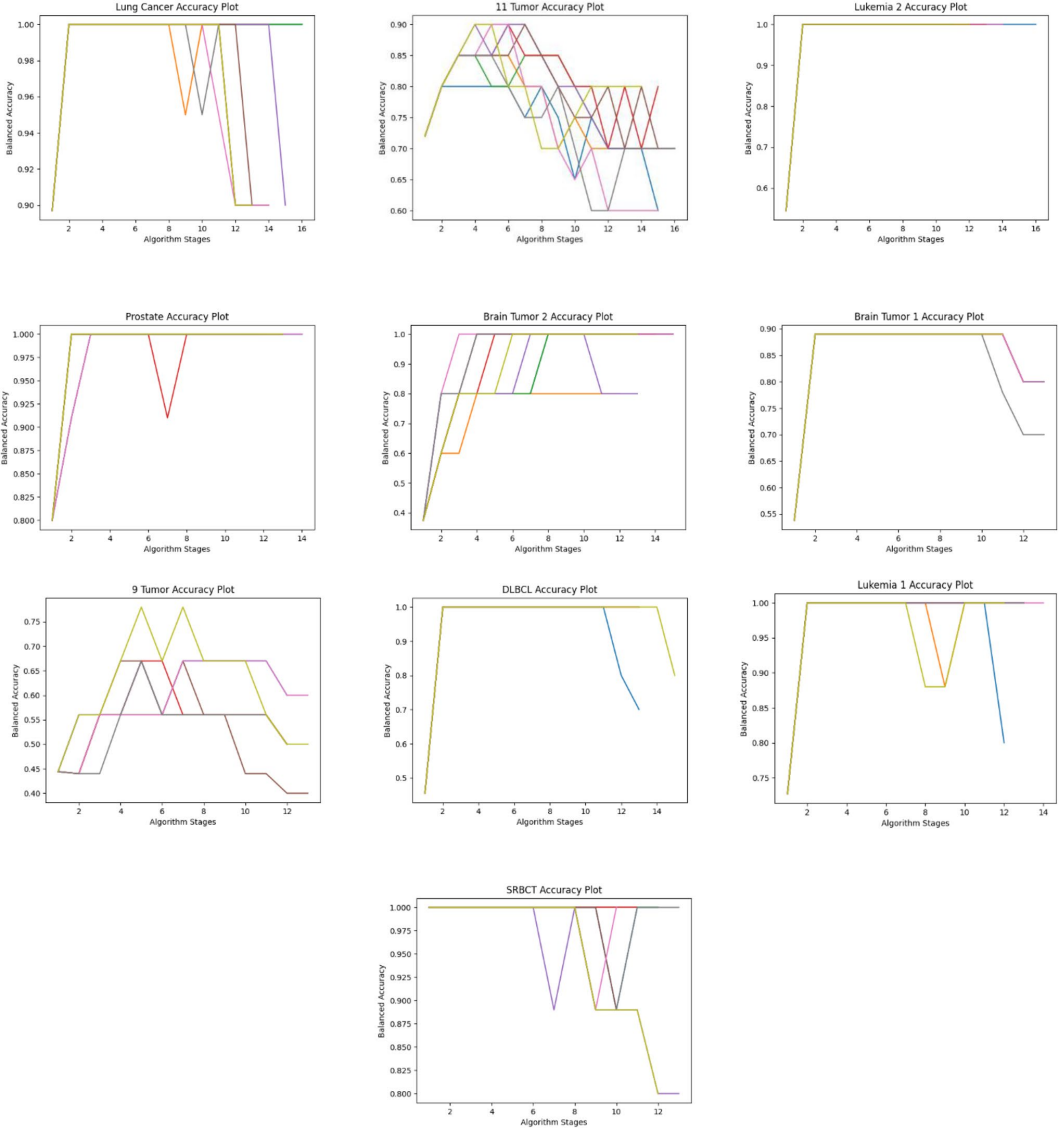
Accuracy of the proposed algorithm on ten different datasets over ten independent runs. The graphs show the improving classification accuracy with recursive stages.

The dataset size reduction process is implemented iteratively until the accuracy and feature count remain consistent in three consecutive stages. During these initial stages, the dimensionality reduction is carried out without sacrificing precision. In the following three stages, the criteria for maintaining accuracy are relaxed, allowing for further reduction in dimensions with the possibility of fluctuating accuracy. Figures 16 and 17 summarize the results of 10 separate runs on all datasets using these additional stages. It can be seen that the number of features decreases as the stages progress. Initially, accuracy increases consistently, but in the last three stages, accuracy may decline as the feature count decreases. The results show that, while the balanced accuracy may vary among the same dataset experiments in the early stages, it eventually converges to a similar level in the later stages.

**Figure 18:**
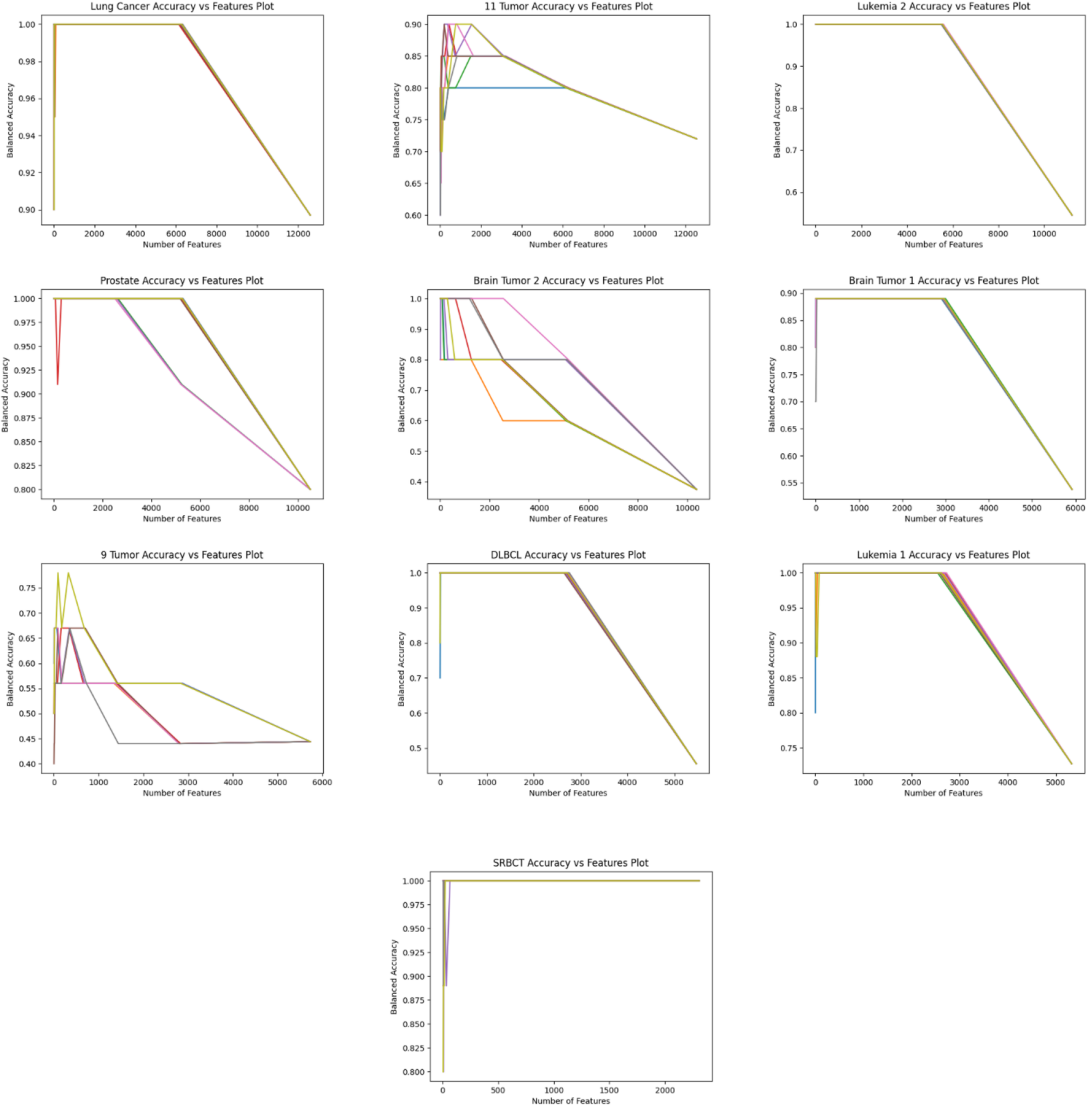
The performance regarding the number of features versus Balanced accuracy. The representation demonstrates the effectiveness of reducing dimensionality over all ten datasets.

Additionally, Fig. 18 demonstrates that as the feature count decreases, the balanced accuracy for all datasets improves, highlighting the critical role of feature selection in attaining optimal accuracy and its potential for reducing the actual feature vector size. It is noteworthy that there is a trade-off between the number of features and accuracy, as reducing the feature vector size too much can result in decreased accuracy in most cases.

To showcase the versatility of our approach, we expanded our analysis by incorporating two additional classification models, Random Forest and Support Vector Machine (SVM), in addition to the KNN model. We conducted experiments on two datasets, ‘brain tumor 1’ and ‘brain tumor 2’, to assess the accuracy of the BEV and Autoencoder algorithms. We evaluated and compared the performance of these algorithms by averaging the results obtained from 10 experiments. These datasets were intentionally selected as they offer potential for improvement beyond what the BEV algorithm achieves in terms of accuracy. The corresponding comparison is presented in Table 6. Details of the Autoencoder parameters used for these evaluations can be found in Table 7.

**Table 6:** Accuracy comparison of BEV and Autoencoder algorithms on two datasets: ‘brain tumor 1’ and ‘brain tumor 2’ based on the average of 10 experiments.

Datasets	Brain tumor 1		Brain tumor 2	
Models	Proposed BEV	Autoencoder	Proposed BEV	Autoencoder
KNN	**89%**	73%	**98%**	74%
Random Forest	**89%**	62.20%	**100%**	52%
SVM	**89%**	65%	**95%**	2%
Best features	**7**	100	**5**	100

Significant values are in bold.

**Table 7:** Autoencoder parameters.

Autoencoder parameters	Values
Optimizer	Adam
Loss	Mean squared error
Epochs	1000, 2500, 5000
Batch size	32, 64, 128
Desired features	7, 50, 100

The results clearly demonstrate that our proposed algorithm outperforms the Autoencoder when employing different classification models on the aforementioned datasets. Notably, the BEV algorithm achieves optimal performance by selecting only 7 and 5 features for ‘brain tumor 1’ and ‘brain tumor 2’, respectively, whereas the Autoencoder attains its best performance with 100 features on both datasets.

Moreover, our proposed model offers a distinct advantage by eliminating the need for a predefined number of desired feature selections, which is a requirement in the Autoencoder approach. In order to investigate the influence of desired feature selection on the Autoencoder’s performance, we conducted experiments utilizing various feature configurations on the ‘Brain Tumor 1’ and ‘Brain Tumor 2’ datasets.

To ensure a fair comparison, we specifically examined the performance of two feature sets: one with 7 features for the ‘brain tumor 1’ dataset and another with 5 features for the ‘brain tumor 2’ dataset. These feature sets represent the average number of features obtained by the BEV algorithm for each dataset. Additionally, we assessed the performance of the Autoencoder using two different desired feature settings: 50 and 100 features. The performance of the Autoencoder under these settings for the two datasets is presented in Table 8, based on the average results from 10 experiments. These analyses allow us to explore the impact of feature selection on the Autoencoder's performance.

**Table 8:** Autoencoder performance on different desired features on two datasets i.e., ‘brain tumor 1’ and ‘brain tumor 2’ based on AVG of 10 experiments.

Autoencoder performance	Brain tumor 1 (%)			Brain tumor 2 (%)		
Models	7 feats	50 feats	100 feats	5 feats	50 feats	100 feats
KNN	55.50	**67.70**	**73.30**	**70**	**67.9**	**74**
Random forest	**60.00**	62.20	57.70	52	46	38
SVM	59.0	59.0	65.50	0	0	2

Significant values are in bold.

After analyzing the results, we made several key observations. Firstly, the random forest classifier demonstrated the best performance when utilizing the autoencoder with 7 features. However, when employing 50 and 100 features, the KNN classifier outperformed other classification models. It is important to highlight that, despite the varying performance across different feature configurations, none of the results surpassed the accuracy and feature efficiency achieved by the BEV algorithm.

Furthermore, we emphasize that the BEV algorithm excels in extracting precise features, ensuring the preservation of the exact features present in the dataset. In contrast, the autoencoder learns compressed representations that may not directly align with the original features of the data. This distinction highlights the strength of the BEV algorithm in capturing relevant information from the dataset.

### Comparison with existing literature

**Figure 19:**
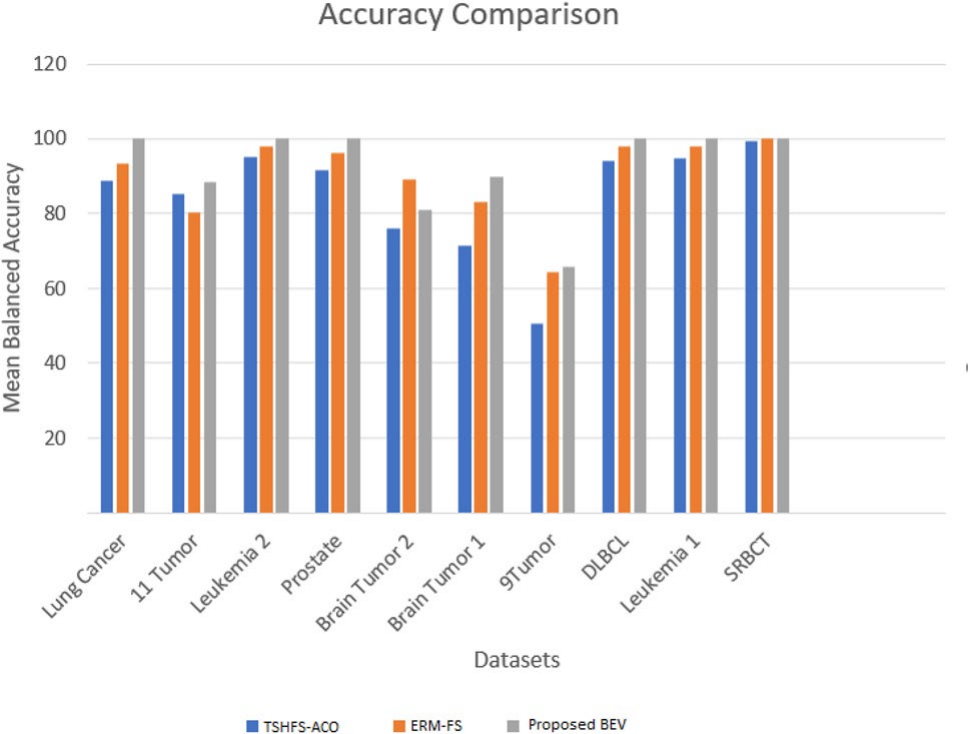
Comparison of proposed BEV with best performing TSHFS-ACO and ERM-FS in terms of Mean Balanced Accuracy (%) on all the datasets. The datasets are ranked in numbers from the highest dimensions to the lowest dimensions.

Table 9 presents the results of the proposed methodology against state-of-the-art approaches in terms of balanced classification accuracy. The proposed BEV method outperforms current state-of-the-art techniques, including the two best methods TSHFS-ACO and ERM-FS, in balanced classification accuracy. BEV achieved an average improvement of 9.21% and 4.23% over TSHFS-ACO and ERM-FS, respectively. The largest improvement was observed in the Brain Tumor 2 dataset, with 8.77% and 21.92% over ERM-FS and TSHFS-ACO. The second largest improvement was seen in Brain Tumor 1 dataset, with 5.74% and 17.58% improvement, respectively. The lowest improvement was 1.88% and 5% on Leukemia 2 dataset. The proposed method performed best in the largest dataset, Lung Cancer (with 12,600 dimensions), with 11.38% and 6.75% improvement over TSHFS-ACO and ERM-FS, respectively (see Table 9). Figure 19 highlights the superiority of our approach in comparison with the two best techniques TSHFS-ACO and ERM-FS in terms of accuracy.

**Table 9:** Comparison in terms of average balanced accuracy with existing studies in 100 feature selection runs (mean ± std).

Dataset	Method									Proposed BEV
	FSBACOM	TFSACO	IRRFSACO	TSHFS-ACO	PSO	ECLPSO	CSO	VLPSO	ERM-FS	
Lung cancer	82.81 ± 1.54	88.84 ± 2.24	86.67 ± 3.13	88.62 ± 0.84	78.77 ± 1.53	77.91 ± 1.98	87.72 ± 2.93	87.60 ± 1.20	93.25 $\pm$ 0.01	**100.0 **$\pm$ **0.00**
11 tumor	80.21 ± 1.76	81.71 ± 1.17	78.85 ± 1.60	85.12 ± 1.30	71.81 ± 1.75	71.09 ± 1.20	79.52 ± 2.35	82.38 ± 1.94	80.26 $\pm$ 0.02	**87.00** $\pm$ **3.49**
Leukemia 2	92.22 ± 2.12	92.44 ± 2.04	87.72 ± 2.44	95.00 ± 1.43	89.83 ± 1.00	89.82 ± 1.20	91.71 ± 3.16	91.56 ± 2.05	98.12 $\pm$ 0.01	**100.0** $\pm$ **0.00**
Prostate	83.98 ± 1.90	88.73 ± 2.02	91.05 ± 2.31	91.57 ± 1.21	86.00 ± 1.49	85.46 ± 1.41	88.99 ± 2.68	88.48 ± 1.93	96.12 $\pm$ 0.01	**100.0** $\pm$ **0.00**
Brain tumor 2	67.21 ± 4.48	72.33 ± 3.29	72.58 ± 4.58	76.08 ± 3.68	61.99 ± 2.91	63.20 ± 2.60	80.44 ± 6.28	70.29 ± 5.25	89.23 $\pm$ 0.03	**98.00** $\pm$ **5.37**
Brain tumor 1	74.83 ± 1.78	74.04 ± 3.33	63.88 ± 4.53	71.42 ± 3.97	73.73 ± 2.21	73.87 ± 2.37	79.93 ± 3.09	70.58 ± 2.78	83.26 $\pm$ 0.04	**89.00** $\pm$ **0.00**
9 tumor	40.83 ± 5.49	45.83 ± 5.33	40.00 ± 3.80	50.67 ± 5.53	42.72 ± 1.42	41.33 ± 1.48	59.50 ± 3.72	47.33 ± 4.23	64.44 $\pm$ 0.07	**64.80** $\pm$ **7.04**
DLBCL	89.48 ± 2.00	91.22 ± 6.43	91.53 ± 3.66	93.95 ± 1.68	83.67 ± 1.52	82.44 ± 2.01	94.30 ± 4.05	91.03 ± 3.85	98.09 $\pm$ 0.02	**100.0** $\pm$ **0.00**
Leukemia 1	84.68 ± 4.23	93.90 ± 1.13	80.32 ± 2.78	94.81 ± 2.35	80.60 ± 2.55	80.88 ± 2.28	88.45 ± 3.90	96.05 ± 2.62	97.93 $\pm$ 0.01	**100.0** $\pm$ **0.00**
SRBCT	88.96 ± 3.00	98.71 ± 0.89	89.13 ± 2.35	99.42 ± 0.53	89.51 ± 1.56	88.10 ± 1.57	93.29 ± 5.52	98.88 ± 0.70	100 $\pm$ 0.00	**100.0** $\pm$ **0.00**

Significant values are in bold.

Table 10 compares the average number of selected features for various techniques. Despite a higher mean balanced accuracy, the proposed BEV approach results in a lower average number of selected features on 8 out of 10 datasets. This highlights the efficiency of the proposed BEV in identifying the optimal features while reducing dimensions. The TFSACO performed better in reducing dimensions on 2 out of 10 datasets. Table 11 presents a comparison of the average balanced accuracy and the average number of selected features of classical studies. It clearly shows that the proposed BEV approach outperforms all other techniques in overall performance. In conclusion, these results demonstrate the superiority of the proposed BEV for high-dimensional feature selection.

**Table 10:** Comparison in terms of average number of features selected with existing studies in 100 feature selection runs.

Dataset	Method									Proposed BEV
	PSO	ECLPSO	CSO	FSBACOM	TFSACO	IRRFSACO	TSHFS-ACO	VLPSO	ERM-FS	
Lung cancer	6234.7	5739.7	226.4	379.1	61.9	96.0	96.3	181.0	61.00	**12.1**
11 tumor	6205.0	5731.7	588.6	339.6	**93.7**	146.0	145.9	204.4	292.60	430.6
Leukemia 2	5535.7	5115.6	88.6	363.0	32.2	56.0	55.9	42.7	15.30	**5.6**
Prostate	5193.7	4818.5	357.2	420.9	12.2	65.0	64.89	38.08	11.00	**6.4**
Brain tumor 2	5117.2	4718.7	90.43	218.5	46.8	74.0	74.1	113.6	26.80	**6.5**
Brain tumor 1	2917.2	2710.0	207.6	120.5	38.7	71.0	70.9	103.8	32.44	**7.3**
9 tumor	2811.9	2605.5	220.3	123.9	**79.9**	89.0	89.2	87.6	89.40	108
DLBCL	2681.0	2491.3	30.1	173.9	17.4	45.0	45.0	31.9	14.20	**6.0**
Leukemia 1	2615.5	2427.9	170.1	130.1	37.8	45.0	44.5	7.0	22.80	**6.1**
SRBCT	1119.4	1054.8	85.4	35.8	49.8	43.0	42.5	23.4	31.00	**10.4**

Significant values are in bold.

**Table 11:** Comparison in terms of average balanced accuracy and number of selected features with classical studies in 100 feature selection runs.

Dataset	Average balanced accuracy				Proposed BEV	Average number of selected features				Proposed BEV
	LFS	CFS	FCBF	ERM-FS		LFS	CFS	FCBF	ERM-FS	
Lung cancer	79.62	93.76	92.71	93.25 $\pm$ 0.01	**100 **$\pm$ **0.00**	**8.5**	517.0	439.4	61.00	12.1
11 Tumor	61.76	80.04	80.57	80.26$\pm$ 0.02	**87.00 **$\pm$ **3.49**	**17.3**	361.6	349.6	292.60	430.6
Leukemia 2	89.44	94.44	95.56	98.12$\pm$ 0.01	**100** ± **0.00**	**4.7**	129.5	77.5	15.30	5.6
Prostate	90.17	92.17	92.17	96.12 ± 0.01	**100** ± **0.00**	**5.9**	80.4	66.1	11.00	6.4
Brain tumor 2	77.50	77.50	77.50	89.23 ± 0.03	**98.00** ± **5.37**	9.1	101.1	66.2	26.80	**6.5**
Brain tumor 1	63.33	76.6.7	73.75	83.26 ± 0.04	**89.00** ± **0.00**	12.2	151.9	104.6	32.44	**7.3**
9 tumor	26.67	56.67	55.00	64.44 ± 0.07	**64.80** ± **7.04**	**9.7**	44.0	33.7	89.40	108
DLBCL	83.33	93.00	94.83	98.09 ± 0.02	**100** ± **0.00**	**5.9**	86.3	66.1	14.20	6.0
Leukemia 1	85.14	92.08	89.86	97.93 ± 0.01	**100** ± **0.00**	**5.4**	79.4	48.5	22.80	6.1
SRBCT	91.67	99.17	98.75	100 ± 0.00	**100** ± **0.00**	**7.1**	112.3	69.0	31.00	10.4

Significant values are in bold.

To assess the performance of the BEV algorithm, we conducted an evaluation with recall which is an important metric in addition to accuracy. We compared the results of the BEV algorithm with the ERM-FS algorithm, which achieved the second-highest accuracy after our proposed algorithm, as shown in Table 11. The evaluation was performed using 'macro' recall since our scenario involved multiple classes. The results for both algorithms can be found in Table 5. It is important to note that 'macro' recall was utilized to ensure a comprehensive evaluation in our multi-class setting.

Table 12 demonstrates that the BEV algorithm consistently surpasses the ERM-FS algorithm in terms of macro recall across the various datasets. This finding highlights the superior performance of the BEV algorithm in accurately capturing important information from the data. In fact, the BEV algorithm achieves a perfect macro recall score of 100% on the majority of the datasets, further emphasizing its effectiveness. However, it is important to mention that in the case of 11 Tumor, Brain Tumor 1, and 9 Tumor datasets, the BEV algorithm exhibits a comparatively lower macro recall of 86.3%, 80%, and 66.6% respectively, indicating an area with potential for improvement.

**Table 12:** Macro recall comparison of ERM-FS and BEV algorithms on multiple datasets.

Datasets	ERM-FS recall	BEV recall
Lung cancer	93.5	**100**
11 tumor	**88.4**	86.3
Leukemia 2	92.7	**100**
Prostate	81.7	**100**
Brain tumor 2	93.1	**100**
Brain tumor 1	89.4	80
9 tumor	43.62	**66.6**
DLBCL	91.1	**100**
Leukemia 1	91.42	**100**
SRBCT	87.8	**100**

Significant values are in bold.

### Algorithm complexity

The BEV algorithm utilizes the KNN model as its classification model. During training, the time complexity of the KNN model is $O(1)$, indicating that it does not depend on the size or dimensionality of the dataset. However, during prediction, the time complexity becomes $O(k\cdot n\cdot d)$, where $k$ represents the number of neighbors, $n$ denotes the number of samples/points in the data, and $d$ represents the dimensionality of the dataset. It's important to note that the time required for distance calculations is typically insignificant compared to other algorithmic steps. The performance of the BEV algorithm is primarily affected by the dimensionality of the dataset. As the dimensionality increases, the computational time also increases. Consequently, the overall time complexity of the BEV algorithm can be expressed as $O({d}^{2}\cdot n)$, assuming the number of neighbors ($k$) remains constant. Table 4 provides the computational time needed for the different algorithms. The algorithms were executed on an Intel Core i7-4770 CPU @3.4 GHz.

According to Table 13, the proposed algorithm is positioned as the third fastest in terms of average computation time across all datasets. It is noteworthy that VLPSO exhibits the highest speed, followed by ERM-FS. However, it is important to emphasize that although VLPSO excels in computational efficiency, it does not rank among the top algorithms in terms of accuracy. Conversely, the proposed algorithm demonstrates slightly slower computation time compared to ERM-FS, but it achieves significantly better accuracy performance while utilizing a reduced number of features.

**Table 13:** Computational time comparison of various algorithms*.*

Datasets	Dimensions	Time (min)					Proposed BEV
		PSO	ECLPSO	CSO	VLPSO	ERM-FS	
Lung cancer	12,600	574.2	503.1	5565.9	70.1	30.33	87.37
11 tumor	12,533	418.5	366.7	6288.6	65.8	37.18	78.95
Leukemia 2	11,225	120.6	125.6	1845.2	16.9	33.19	47.28
Prostate	10,509	160.6	152.5	2369.9	22.6	29.1	45.14
Brain tumor 2	10,367	80.5	73.6	950.8	12.1	27.39	40.14
Brain tumor 1	5920	66.7	60	462.1	9.8	15.45	21.88
9 tumor	5726	39.2	39.2	373.4	6.2	15.53	20.35
DLBCL	5469	47.6	44.2	394.8	7.4	14.46	19.89
Leukemia 1	5327	41.2	36.3	251.8	6.4	12.08	19.12
SRBCT	2308	8.2	7.5	19.9	1.4	6.82	8.61
Average time (min)		155.7	140.9	1852.2	21.9	22.2	38.9

## Conclusion

The proposed Bird's Eye View (BEV) feature selection approach offers a solution to the challenge of selecting features in high-dimensional datasets. It combines three different paradigms and employs a rewarding scheme and collective evolution with Markov impact to iteratively reduce the feature space. The BEV algorithm draws inspiration from the genetic algorithm mechanism and implements a smart branching evolution approach that relies on dynamic Markov chains. The algorithm begins by initializing a root leaf and proceeds to generate children leaves, where the number of generated leaves is determined by a predetermined fixed value. Each leaf is represented by a sequence of 1 s and 0 s, organized in pairs. The best leaves are selected for each expansion based on evaluation. This iterative process continues until no further improvement is observed. The BEV algorithm effectively distinguishes between different classes by utilizing a reward and penalty mechanism to update transition probabilities during state transitions. This mechanism is based on the improvement or lack thereof in the fitness function. As a result, the algorithm achieves a significantly reduced feature subset while preserving high classification performance.

The effectiveness of the proposed BEV approach in high-dimensional feature selection is demonstrated by its ability to generate a significantly reduced feature subset while maintaining a high fitness level. Through evaluation on 10 benchmark datasets, the BEV model outperforms current state-of-the-art methods. Furthermore, it offers advantages such as simplicity in development, ease of hyperparameter configuration, and fast execution.

However, it is important to note that our approach is a stochastic algorithm, which means it provides suboptimal solutions rather than guaranteed optimal solutions. Despite effectively exploring the search space, there is no guarantee that the selected feature subset will be the absolute best. Achieving satisfactory performance in the proposed approach depends heavily on fine-tuning various hyperparameters. One avenue for future research involves exploring the tuning of additional hyperparameters to enhance the algorithm's performance. Additionally, we plan to investigate the inclusion of sets of k-features, as opposed to limiting the selection to only two features. This modification aims to assess whether expanding the feature selection scope can further improve the approach's performance.

## Data Availability

The code and datasets are available from the links https://github.com/Bilal39/Bird-Eye-View-Script and https://github.com/tnbinh/VLPSO/tree/main/Data.
